# Curating maternal, neonatal and child health (MNCH) datasets for spatiotemporal data analytics

**DOI:** 10.12688/f1000research.73822.1

**Published:** 2022-02-10

**Authors:** Moses Effiong Ekpenyong, Patience Usoro Usip, Kommomo Jacob Usang, Nnamso Michael Umoh, Samuel Bisong Oyong, Chukwudi Obinna Nwokoro, Aminu Alhaji Suleiman, Kingsley Attai, Anietie Emmanuel John, Inyang Abraham Clement, Ekemini Anietie Johnson, Temitope Joel Fakiyesi

**Affiliations:** 1 Department of Computer Science, University of Uyo, Uyo, Akwa Ibom, 520003, Nigeria; 2 Centre for Research and Development, University of Uyo, Uyo, Akwa Ibom, 520003, Nigeria; 3 Department of Computer Science, Abdu Gusau Polytechnic, Gusau, Zamfara, Nigeria; 4 Department of Computer Science, Rittman University, Ikot Ekpence, Akwa Ibom, Nigeria

**Keywords:** context-aware system, robust decision support, GeoAI, healthcare indicator, location-based information, MNCH data

## Abstract

We provide in this Data Note the details of maternal, neonatal and child health (MNCH) datasets curated directly from patients’ medical records; comprising 538 maternal, 720 neonatal and 425 child records, captured at St Luke’s General Hospital, Anua, Uyo, Nigeria, from 2014 to 2019. Variables included in the datasets are gender, age, class of patient (mother/infant/child), LGA (local government area), diagnosis, symptoms, prescription, blood pressure (mm Hg), temperature (degree centigrade), and weight (Kg). The purpose of this publication is to describe the datasets for researchers who may be interested in its reuse (for analysis, research, quality assurance, policy formulation/decision, patient safety, and more). The curated datasets also involved the capturing of location information (GPS: global positioning system data) from the study area, to aid spatiotemporal and informed demographic analysis. We detail the methods used to curate the datasets and describe the protocol of variables selection and processing. For reasons of data privacy, some patients’ personal information such as names were replaced with patient numbers (a sequence generated using Microsoft Excel). Furthermore, the addresses/locations of the patients, date of visit, latitude, longitude, elevation, and GPS accuracy are restricted. Restricted data can be made available to readers after a formal request to the corresponding author (see data restriction statement). The curated datasets are available at the Open Science Framework.

## Introduction

Access to health services is essential for promoting health equity and quality of life ( dos Anjos Luis & Cabral, 2016). Hence, knowledge of health facilities is crucial for providing informed health planning decisions. Furthermore, available health datasets such as the one presented in this publication show that some patients travel long distances to access health facilities, with the urban areas holding very high concentration of patients’ population per facility. Oleribe *et al.* (2019) identified financial barrier, poor governance and limited infrastructure, as major factors mitigating access to quality healthcare, typical of sub-Saharan Africa. Situating these factors to the patients’ domain in a country such as Nigeria, reveals the following mitigating factors: finance (poor living condition), increased security threat, type and nature of ailment, geography of residence, race and ethnicity, gender, age, language, and disability. These factors significantly determine the degree at which medical/healthcare services – including availability, timeliness, convenience, and affordability, are utilised ( Babalola & Fatusi, 2009). Adoption of modern technology has however simplified healthcare services through the implementation of automated systems. For instance, the integration of electronic health records and predictive intelligence (e.g., smart technology) into healthcare services have achieved efficient, accurate storage and retrieval of patients’ records, as well as intelligent data-driven analysis, prediction, and visualisation ( Tian *et al.*, 2019).

Unlike developed nations, health facilities in low- and medium-income countries such as the sub-Sahara African region are overly stressed, generating a large pool of manually unstructured and inconsistent data that defies real-time, patient-centred care. Furthermore, the reduced health budget has decreased government’s efforts in establishing new healthcare centres to equate the present population growth, hence, increasing the establishment of privately owned healthcare centres, premised on business and which services are not conducive to patients in terms of cost. Even though availability of health facilities is often prioritised over accessibility by decision makers ( Tuba *et al.*, 2010), Mishra *et al.* (2019) maintained that geographic accessibility and availability of healthcare facilities are essential parameters in determining the quality of care received, as analysis of both parameters could reveal useful patterns and trends for providing a more robust health system that derives patient-centred care. Patient-centred care ( Epstein & Street, 2011) empowers patients to actively participate in their care with physicians and other healthcare providers connecting with patients to effectively address patients’ needs. In providing such a service, location-based information, and details of every parameter within the health system should be available in real-time. Also, collaboration between the necessary stakeholders (physicians and other healthcare providers, government, patients) is necessary and can be enabled using geospatial artificial intelligence (GeoAI) ( VoPham *et al*., 2018; Boulos *et al*., 2020). GeoAI combines spatial science methods such as GIS (geographic information system), data mining, and high-performance computing to extract meaningful knowledge from spatial big data ( VoPham *et al*., 2018; Janowicz *et al*., 2020). By integrating machine learning into ArcGIS, GeoAI offers intelligent context-aware models for accurate location-allocation (e.g., assignment of healthcare facilities) and robust healthcare decisions modelling.

We demonstrate in this publication the importance of unstructured data processing to achieve semi-structured maternal, neonatal and child health (MNCH) datasets curated directly from patients’ medical file/records, to support intelligent health data mining, informed policy planning and robust decision support system design.

The following constitute our expected hypotheses on the applications of our datasets:

- MNCH datasets would encourage the development of resources for the sub-Saharan African region and advance future research progress in the medical/healthcare system of Nigeria.
- MNCH datasets would drive integrated GeoAI applications for robust spatiotemporal data analysis.
- MNCH datasets would support decision systems with suitable ontologies or knowledge representations, to drive intelligent data analytics.

A study using the MNCH datasets is Usip *et al.* (2021), which developed a preposition-enabled parser that extracts prepositions from clinical notes to visualise unstructured patients’ data using generated location items such as noun phrase, geolocation, and place name.

## Materials and methods

### Ethical approval

Ethical clearance was granted by the University of Uyo Health Research Ethics Committee (UNIUYO-HREC) – Ref. number: UU/CHS/IHREC/VOL.I/017 with the acceptance that the study did not require direct contact with patients.

### Data source, sample size and capturing procedure

The source of the datasets is patients’ medical records/files retrieved from the St Luke’s General Hospital, Anua, Uyo, Akwa Ibom State, Nigeria (the healthcare facility), between 2014 and 2019. The study subjects were randomly selected through physical inspection of the patients’ records (or file bundles) manually stored in the hospital’s file cabinets or archives. To facilitate this exercise, informed consent was processed through the Chief Medical Director of the hospital, to retrieve the required data. This permission was granted, and logistics were put in place to commence the exercise. The investigators did not have access to the records room or (patients’ population) archive. Only files selected by the responsible officers assigned by the Chief Medical Director of the hospital were presented to the investigators. Before giving the files to the investigators for attributes capture, the officers glossed through the files to ensure that the primary attributes of the study (see Table 1) are satisfied.

**Table 1.** Description of maternal, neonatal and child health (MNCH) data capture template.

Attribute	Description
Date of visit	Date patient visited the hospital
Gender	Gender of patient
Age	Age of patient
Class of patient	Age classification (Mother, Infant or Child)
Address	Home address or location of the patient
Symptom	The cause of the ailment
Diagnosis	Outcome of the examination of patient
Prescription	Administered therapy/drug
Health history	Health history of patient
Health status	Health status of patient (Apgar score), ( Finster *et al.*, 2005)
Blood pressure	Blood pressure of patient in millimetre per mercury (mm/Hg)
Temperature	Temperature of the patient in degree centigrade (°C)
Height	Height of patient in centimetres (cm)
Weight	Weight of patient in kilogrammes (Kg)

To capture the primary attributes for maternal, neonatal and child health, a data template (a table with attributes of the study), was designed with ethical considerations in mind. No sensitive information such as patient’s name was captured. Furthermore, patient’s location/address was truncated by removing street number, to avoid privacy and security implication. Maternal health data template had the following attributes (Date of visit, Gender, Class of patient [mother/infant/child], Address, Symptom, Diagnosis, Prescription, Blood pressure, Temperature, Weight). Neonatal health data template had the following attributes (Date of visit, Gender, Age, Class of patient, Address, Symptom, Condition, Height, Weight). Child health data template had the following attributes (Date of visit, Gender, Age, Class of patient, Address, Diagnosis, Health history, temperature, Weight). The description of these attributes is tabulated on Table 1.

The total sample of data retrieved (before processing) included maternal (1063), neonatal (1367) and child patients (826), covering the 3 senatorial districts of Akwa Ibom State namely Uyo, Ikot Ekpene and Eket, and the 31 local government areas (LGAs) as presented on Table 2.

**Table 2.** Senatorial districts and local government areas of captured data. LGA=local government area.

Senatorial Districts	LGA	Number of LGA
Uyo	Uyo, Itu, Uruan, Etinan, Ibiono Ibom, Nsit Ibom, NsitUbium, Nsit Atai, Ibesikpo Asutan	9
Eket	Eket, Ikot Abasi, Mkpat Enin, ONNA, Eastern Obolo, Esit Eket, Ibeno, Okobo, Mbo, Oron, Udung Uko, Urue Offong Oruko	12
Ikot Ekpene	Ikot Ekpene, Abak, ObotAkara, Ika, Ukanafun, Etim Ekpo, Ini, Ikono, Oruk Anam	10
	Total:	31

### Geolocation capture and data processing

To enable the support of GeoAI services, additional attributes were collected by visiting the respective study locations. The visited locations were those associated with the collected data. The UTM Geo Map, a simple android application for coordinates capture, GIS, and Spatial analysis was deployed for this purpose. The UTM Geo Map app can be downloaded from the Google play store, and has several modules, but the Map Coordinates module, which maps coordinates in real-time was used to capture the respective location coordinates. The process for obtaining the location coordinates (latitude and longitude) using the UTM Geo Map app are summarised as follows:

Step 1: Launch the UTM Geo Map app when in the vicinity of patient address
Step 2: Select *Map Coordinates*
Step 3: Select *Goto GPS Location* (this step gives the real-time location of the mobile device with GPS accuracy in meters appearing on the screen. Ensure that the GPS accuracy is within an acceptable range).
Step 4: Select *Mark*. A request to enter the Point Name will pop up. Enter the point name or address of the patient
Step 5: Select *Save*. Each saved point is stored on the mobile device. To transfer the measured data to an external file, there is an *Export tool*, which supports different file formats such as KML, CSV, GPX, DXF, TXT, GeoJSON.
Step 6: Select *Export/Import*, *Export to CSV*, type in a filename with “.csv” extension.
Step 7: Select *Save*.

The exported file format used in this publication is the CSV format, and the columns (attributes) extracted are described in Table 3. A GPS accuracy range of 1 – 9.65 metres (i.e., how close the device’s calculated position is from the truth, expressed as a radius), was used as an acceptable accuracy range for this publication. A lower GPS accuracy defines the precision of the patient location. The coordinates capturing was carried out by doctoral students, using different mobile devices. Where the GPS accuracy was too high, such location was recaptured and tuned to the acceptable accuracy range. Due to ethical reasons, we are only interested in the vicinity of the patient, hence the defined accuracy range. 

**Table 3.** Extracted attributes of location coordinates.

Attribute	Description	Sample data	Data type
ID	Identity or point	PT_4 Etuk Allan street itam	Alphanumeric
Latitude	Latitude is the angle ranges from 0° at the Equator to 90° (North or South) at the poles	5.0437963	Numeric
Longitude	Longitude is the measurement east or west of the prime meridian (0–180°) East or west	7.8936366	Numeric
Notes	Descriptive	Null	Alpha
DMS	Degrees, minutes, and seconds	5Â° 2’ 37.67’’ N | 7Â° 53’ 37.09’’ E	Alphanumeric
UTM	Universal Transverse Mercator	377355.436E557609.59N32N	Alphanumeric
MGRS	Military Grid Reference System	32NLL 77355 57610	Alphanumeric
CRS	Coordinate Reference System	7.8936366 5.0437963	Numeric
CRS Code	Coordinate Reference System code	EPSG:4326	Alphanumeric
Elevation (MSL)	Elevation of Mean Sea Level	69.46	Numeric
Address	Location	Null	Alphanumeric
Date Record	Capture date	Record Date: 2021-05-20 11:11:02	Numeric
GPS Accuracy (m)	Global Positioning System (GPS) Accuracy	3.900000095	Numeric
Photo	Picture of the location	Null	Image

To clearly mark the location boundaries of patients and geographically localise them within a local government unit, the address column was further split to form an additional attribute, called the LGA. Location attributes documented as part of the datasets include Latitude, Longitude, Elevation, Date recorded, and GPS accuracy. For this publication, we were only able to provide location data for patients within the Uyo metropolis, hence, resulting in a total of 1683 MNCH records and distributed as follows: maternal=538, neonatal=720, child=425. We hope to cover other senatorial districts as soon as future funding is available.

**Figure 1.**
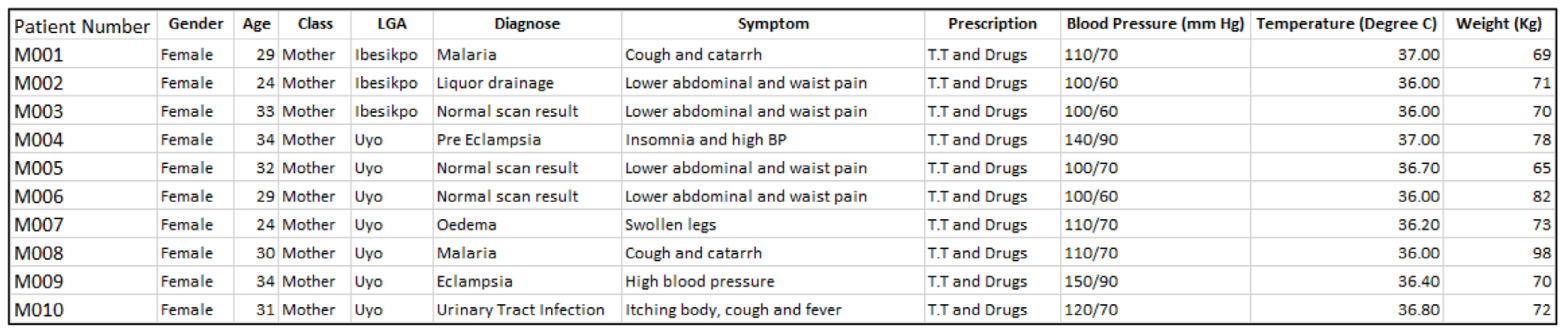
Sample maternal health dataset. LGA=local government area.

**Figure 2.**
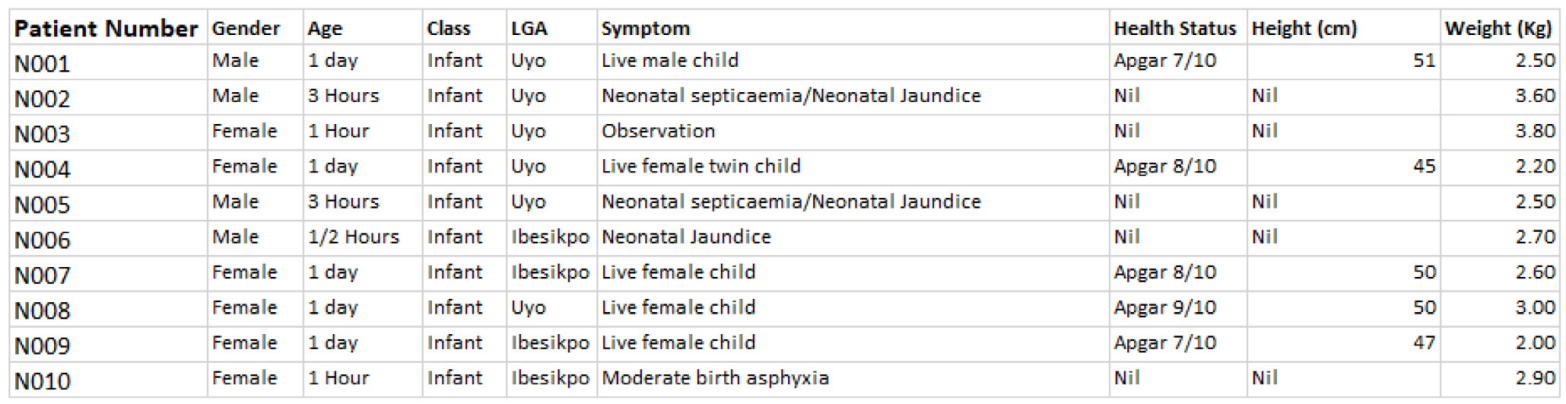
Sample neonatal health dataset. LGA=local government area.

**Figure 3.**
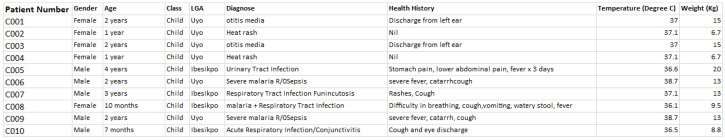
Sample child health dataset. LGA=local government area.

At the end of the data capturing exercise, the data template was converted into electronic format using Microsoft Excel, and manually merged with the geolocation records (exported CSV file) from the field (or study locations visited). The first 10 samples of the maternal, neonatal and child health datasets are given in Figure 1, Figure 2, and Figure 3, respectively. The dataset can be found as *Underlying data* ( Ekpenyong *et al.*, 2021).

## Data availability

### Underlying data

Open Science Framework: Maternal, Neonatal and Child Health Datasets for Spatiotemporal Data Analytics. https://doi.org/10.17605/OSF.IO/J9ZH8 ( Ekpenyong *et al.*, 2021).

Data are available under the terms of the Creative Commons Attribution 4.0 International license (CC-BY 4.0).

### GPS data

Access to restricted data (GPS data) will be made available to readers after a formal request to the corresponding author ( mosesekpenyong@uniuyo.edu.ng) and on the condition that data will be used strictly for research purposes.
