# Curating maternal, neonatal and child health (MNCH) datasets from a hospital’s catchment area in Nigeria between 2014 and 2019

**DOI:** 10.12688/f1000research.73822.2

**Published:** 2023-09-11

**Authors:** Moses Effiong Ekpenyong, Patience Usoro Usip, Kommomo Jacob Usang, Nnamso Michael Umoh, Samuel Bisong Oyong, Chukwudi Obinna Nwokoro, Aminu Alhaji Suleiman, Kingsley Attai, Anietie Emmanuel John, Inyang Abraham Clement, Ekemini Anietie Johnson, Temitope Joel Fakiyesi

**Affiliations:** 1 Centre for Research and Development, University of Uyo, Uyo, Akwa Ibom, 520003, Nigeria; 2 Department of Computer Science, University of Uyo, Uyo, Akwa Ibom, 520003, Nigeria; 3 Department of Computer Science, Abdu Gusau Polytechnic, Gusau, Zamfara, Nigeria; 4 Department of Computer Science, Rittman University, Ikot Ekpence, Akwa Ibom, Nigeria

**Keywords:** context-aware system, robust decision support, GeoAI, healthcare indicator, location-based information, MNCH data

## Abstract

In this Data Note, we present details regarding Maternal, Neonatal, and Child Health (MNCH) datasets sourced directly from patients' medical records. These datasets consist of 538 maternal, 720 neonatal, and 425 child records, all collected at St Luke’s General Hospital in Anua, Uyo, Nigeria, spanning from 2014 to 2019. Variables included in the datasets are: Maternal {patient number, date of visit, gender, age, class of patient, address, LGA, diagnose, symptom, prescription, blood pressure (mm Hg), temperature (degree centigrade), weight (Kg), latitude, longitude, elevation, (MSL), date record, GPS Accuracy (m)}; Neonatal {patient number, date of visit, gender, age, class of patient, address, LGA, symptom, health status, height (cm), weight (Kg), latitude, longitude, elevation (MSL), date record, GPS Accuracy (m)}; and Child Health {patient number, date of visit, gender, age, class of patient, address, LGA, diagnose, health history, temperature (degree centigrade), weight (Kg), latitude, longitude, elevation (MSL), date record, GPS accuracy (m)}. The purpose of sharing these datasets is to provide a resource for researchers interested in their potential reuse, whether for analysis, research, quality assurance, policy formulation, decision-making, patient safety, or other purposes. The datasets also include location information obtained through GPS (Global Positioning System) data from the study area, facilitating spatiotemporal analysis. We outline the methods used for curating the datasets, including the protocol for selecting and processing variables. To protect patient privacy, certain personal details such as names were replaced with unique patient numbers generated using Microsoft Excel. Furthermore, specific patient information, including addresses/locations, date of visit, latitude, longitude, elevation, and GPS accuracy, has been restricted for privacy reasons. Readers interested in accessing restricted data can make a formal request to the corresponding author (see data restriction statement). The curated datasets are available at the Open Science Framework.

## Introduction

Access to health services is essential for promoting health equity and quality of life ( dos Anjos Luis & Cabral, 2016). Hence, knowledge about available MNCH facilities is essential for making informed decisions in health planning. Moreover, available healthcare datasets, like the one presented in this publication, reveal that some patients have to travel long distances to access healthcare facilities. In urban areas, there is a notable imbalance in the patient-to-provider ratio, with a significant concentration of the patient population per healthcare facility. Oleribe *et al*. (2019) identified major factors hindering access to quality healthcare in sub-Saharan Africa, including financial barriers, poor governance, and limited infrastructure. In Nigeria identified hidering factors include, financial constraints due to poor living conditions, heightened security threats affecting healthcare access, the type and nature of ailments influencing healthcare choices, geographic residence impacting access, racial and ethnic disparities affecting healthcare outcomes, gender-related disparities, age-related healthcare considerations, language barriers affecting communication, and disability-related healthcare challenges. These factors significantly influence the utilization of medical and healthcare services in terms of availability, timeliness, convenience, and affordability ( Babalola & Fatusi, 2009). The integration of modern technology into the health sector has simplified healthcare services. For instance, the integration of electronic health records and predictive intelligence (e.g., smart technology) into healthcare services have achieved efficient, accurate storage and retrieval of patients’ records, as well as intelligent data-driven analysis, prediction, and visualisation ( Tian *et al.*, 2019).

Unlike developed nations, health facilities in low- and medium-income countries such as the sub-Sahara African region are overly stressed, generating a large pool of manually unstructured and inconsistent data; defying efforts to extract meaningful insights, hinder accurate decision-making, and impede the creation of reliable healthcare solutions and strategies. Furthermore, the reduced health budget has decreased government’s efforts in establishing new healthcare centres to equate the present population growth, hence, increasing the establishment of privately owned healthcare centres, premised on business and which services are not conducive to patients in terms of cost. Even though availability of health facilities is often prioritised over accessibility by decision makers ( Tuba *et al.*, 2010), Mishra *et al.* (2019) maintained that geographic accessibility and availability of healthcare facilities are essential parameters in determining the quality of care received, as analysis of both parameters could reveal useful patterns and trends for providing a more robust health system that derives patient-centred care. Patient-centred care ( Epstein & Street, 2011) empowers patients to actively participate in their care with physicians and other healthcare providers connecting with patients to effectively address patients’ needs. To achieve this, it's essential that technology-driven systems, aiming to enhance the utility of healthcare delivery systems, prioritize the availability of real-time location-based information and comprehensive details of the healthcare system. Also, collaboration between the necessary stakeholders (physicians and other healthcare providers, government, patients) is necessary and can be enabled using geospatial artificial intelligence (GeoAI) ( Boulos *et al*., 2019; VoPham *et al*., 2018). GeoAI combines spatial science methods such as GIS (geographic information system), data mining, and high-performance computing to extract meaningful knowledge from spatial big data ( Janowicz *et al*., 2020; VoPham *et al*., 2018). 

Geospatial software tools for managing and visualizing MNCH datasets include:

ArcGIS: Developed by Esri (Environmental Systems Research Institute) is a comprehensive and widely used GIS software suite, designed for managing, analyzing, visualizing, and sharing geospatial data and information.

QGIS (Quantum GIS): An open-source and user-friendly GIS software that provides powerful mapping and data analysis capabilities.

GRASS GIS (Geographic Resources Analysis Support System): An open-source GIS software that focuses on geospatial data management, analysis, and visualization.

R with Spatial Packages: The R programming language, coupled with specialized spatial packages like "sp," "sf," and "raster," can be used for geospatial data analysis and visualization.

GeoDa: A free software program designed for exploratory spatial data analysis, spatial statistics, and spatial econometrics.

SAGA GIS: An open-source geographic information system with a wide range of geospatial analysis and modeling tools.

Mapbox: A cloud-based platform for designing and publishing custom maps, providing tools for geospatial data visualization and analysis.

OpenStreetMap (OSM) Tools: Tools and APIs for working with OpenStreetMap data, which can be useful for incorporating community-contributed geographic data.

Google Earth Engine: A cloud-based platform for analyzing and visualizing Earth observation data, which can be valuable for spatiotemporal analysis.

The choice of geospatial software however depends on factors such as, data requirements, project goals, budget, and end-users’ familiarity. Each of these software options offers unique features and capabilities for working with geospatial data.

This publication creates a maternal, neonatal, and child health (MNCH) datasets directly sourced from patients' medical records for a data poor setting. The curated datasets are instrumental in facilitating driving location sensitive decision making, intelligent health data mining, informed policy planning, and robust decision support systems design. The specific objectives of the research therefore include:

- To gather and compile detailed MNCH data from various sources to create a comprehensive dataset.
- To develop a standardized MNCH data and structure format.
- To convert unstructured healthcare data into a semi-structured format, making it suitable for analysis.
- To integrate location-based information into the MNCH dataset for facilitating spatiotemporal analysis and visualization.

The following are the study’s hypothesis:

- Technology driven data curation practices improves MNCH outcome predictions
- Integration of location-based data into MNCH datasets leads to more informed policy insights and recommendations for MNCH.
- Geo-referenced features of MNCH datasets enables real-time demographic/spatiotemporal analysis.

Usip *et al.* (2021) used the MNCH datasets, to develop a parser with preposition recognition capabilities and extract prepositions from clinical notes for unstructured patient data visualization, incorporating generated location items like noun phrases, geolocations, and place names.

## Materials and methods

### Ethical approval

Ethical clearance was granted by the University of Uyo Health Research Ethics Committee (UNIUYO-HREC) – Ref. number: UU/CHS/IHREC/VOL.I/017 with the acceptance that the study did not require direct contact with patients.

### Data source, sample size and capturing procedure

**Figure 1.**
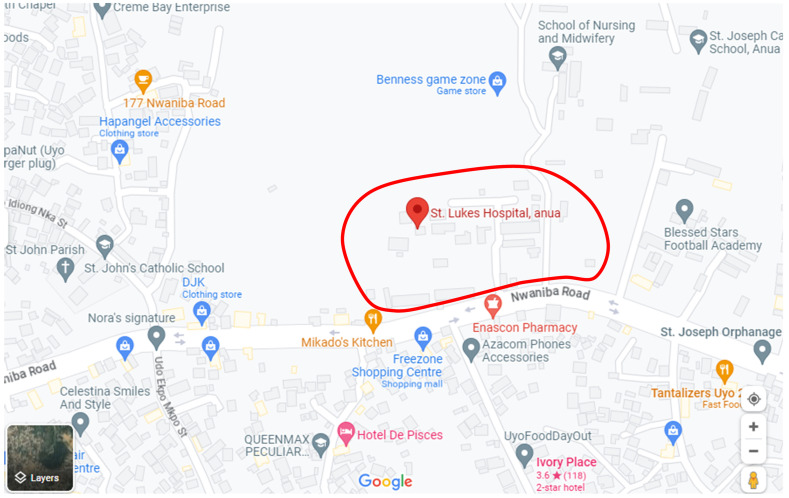
Location map showing St Luke’s General Hospital, Anua.

The source of the datasets is patients’ medical records/files retrieved from the St Luke’s General Hospital, Anua, Uyo, Akwa Ibom State, Nigeria (the healthcare facility). St Luke’s General Hospital, Anua is one of the foremost Missionary Hospitals in the South-South and South-East Nigeria. The hospital is located along Nwaniba Road in Uyo Metropolis of Akwa Ibom State, Nigeria, West Africa. A location map showing the approximate hospital catchment for which the datasets are available is presented in Figure 1.

These records cover the period from 2014 to 2019. The process of selecting properly documented files involved physically inspecting the patients’ records, which were manually stored in the hospital’s file cabinets or archives. To initiate this procedure, we sought informed consent through the Chief Medical Director of the hospital to obtain the necessary data. After obtaining permission, the necessary arrangements were made to commence the exercise. It’s important to note that the investigators did not have direct access to the records room or the patient archives. Only files preselected by the designated officers assigned by the Chief Medical Director of the hospital were made available for the investigators’ use. Before handing over these files for the capture of attributes, the assigned officers reviewed them to ensure compliance with the primary attributes of the study, as outlined in Table 1).

**Table 1.** Description of maternal, neonatal and child health (MNCH) data capture template.

Attribute	Description
Date of visit	Date patient visited the hospital
Gender	Gender of patient
Age	Age of patient
Class of patient	Age classification (Mother, Infant or Child)
Address	Home address or location of the patient
Symptom	The cause of the ailment
Diagnosis	Outcome of the examination of patient
Prescription	Administered therapy/drug
Health history	Health history of patient
Health status	Health status of patient (Apgar score), ( Finster *et al.*, 2005)
Blood pressure	Blood pressure of patient in millimetre per mercury (mm/Hg)
Temperature	Temperature of the patient in degree centigrade (°C)
Height	Height of patient in centimetres (cm)
Weight	Weight of patient in kilogrammes (Kg)

To capture the primary attributes for maternal, neonatal and child health, a data template (a table with attributes of the study), was designed with ethical considerations in mind. Protocols established to maintain the confidentiality and anonymity of patients' health records and to mitigate the risk of inadvertently identifying specific individuals within the local community include:

Data De-Identification: Personal identifiers, such as names, were systematically removed or replaced with codes or pseudonyms to prevent any association between the data and specific individuals.

Location Privacy Measures: Specific location/address details, including street numbers, were redacted or generalized to a broader geographic level to minimize the risk of identifying individual patients based on their addresses.

Aggregated Data Analysis: Instead of analyzing individual-level data, the analysis was conducted at an aggregated or group level whenever possible. This approach ensures that results and insights do not pertain to any single person but are generalized across a broader population.

Access Control: Access to the dataset and any related information was restricted to authorized personnel only, and strict confidentiality agreements were in place to prevent any unauthorized disclosure of patient identities.

Ethical Review and Compliance: The study and its data-handling procedures were subject to ethical review and compliance with relevant privacy and data protection regulations and guidelines to ensure patient anonymity.

Maternal health data template had the following attributes (Date of visit, Gender, Class of patient [mother/infant/child], Address, Symptom, Diagnosis, Prescription, Blood pressure, Temperature, Weight). Neonatal health data template had the following attributes (Date of visit, Gender, Age, Class of patient, Address, Symptom, Condition, Height, Weight). Child health data template had the following attributes (Date of visit, Gender, Age, Class of patient, Address, Diagnosis, Health history, temperature, Weight). The description of these attributes is tabulated on Table 1.

The total sample of data retrieved (before processing) included maternal (1063), neonatal (1367) and child patients (826), covering the 3 senatorial districts of Akwa Ibom State namely Uyo, Ikot Ekpene and Eket, and the 31 local government areas (LGAs) as presented on Table 2.

**Table 2.** Senatorial districts and local government areas of captured data. LGA=local government area.

Senatorial Districts	LGA	Number of LGA
Uyo	Uyo, Itu, Uruan, Etinan, Ibiono Ibom, Nsit Ibom, NsitUbium, Nsit Atai, Ibesikpo Asutan	9
Eket	Eket, Ikot Abasi, Mkpat Enin, ONNA, Eastern Obolo, Esit Eket, Ibeno, Okobo, Mbo, Oron, Udung Uko, Urue Offong Oruko	12
Ikot Ekpene	Ikot Ekpene, Abak, ObotAkara, Ika, Ukanafun, Etim Ekpo, Ini, Ikono, Oruk Anam	10
	Total:	31

### Geolocation capture and data processing

To enable the support of GeoAI services, additional attributes were collected by visiting the respective study locations. The visited locations were those associated with the collected data. The UTM Geo Map, a simple android application for coordinates capture, GIS, and Spatial analysis was deployed for this purpose. The UTM Geo Map app can be downloaded from the Google play store, and has several modules, but the Map Coordinates module, which maps coordinates in real-time was used to capture the respective location coordinates. The process for obtaining the location coordinates (latitude and longitude) using the UTM Geo Map app are summarised as follows:

Step 1: Launch the UTM Geo Map app when in the vicinity of patient address
Step 2: Select *Map Coordinates*
Step 3: Select *Goto GPS Location* (this step gives the real-time location of the mobile device with GPS accuracy in meters appearing on the screen. Ensure that the GPS accuracy is within an acceptable range).
Step 4: Select *Mark*. A request to enter the Point Name will pop up. Enter the point name or address of the patient
Step 5: Select *Save*. Each saved point is stored on the mobile device. To transfer the measured data to an external file, there is an *Export tool*, which supports different file formats such as KML, CSV, GPX, DXF, TXT, GeoJSON.
Step 6: Select *Export/Import*, *Export to CSV*, type in a filename with “.csv” extension.
Step 7: Select *Save*.

The exported file format used in this publication is the CSV format, and the columns (attributes) extracted are described in Table 3. A GPS accuracy range of 1 – 9.65 metres (i.e., how close the device’s calculated position is from the truth, expressed as a radius), was used as an acceptable accuracy range for this publication. A lower GPS accuracy defines the precision of the patient location. The coordinates capturing was carried out by doctoral students, using different mobile devices. Where the GPS accuracy was too high, such location was recaptured and tuned to the acceptable accuracy range. Due to ethical reasons, we are only interested in the vicinity of the patient, hence the defined accuracy range. 

**Table 3.** Extracted attributes of location coordinates.

Attribute	Description	Sample data	Data type
ID	Identity or point	PT_4 Etuk Allan street itam	Alphanumeric
Latitude	Latitude is the angle ranges from 0° at the Equator to 90° (North or South) at the poles	5.0437963	Numeric
Longitude	Longitude is the measurement east or west of the prime meridian (0–180°) East or west	7.8936366	Numeric
Notes	Descriptive	Null	Alpha
DMS	Degrees, minutes, and seconds	5Â° 2’ 37.67’’ N | 7Â° 53’ 37.09’’ E	Alphanumeric
UTM	Universal Transverse Mercator	377355.436E557609.59N32N	Alphanumeric
MGRS	Military Grid Reference System	32NLL 77355 57610	Alphanumeric
CRS	Coordinate Reference System	7.8936366 5.0437963	Numeric
CRS Code	Coordinate Reference System code	EPSG:4326	Alphanumeric
Elevation (MSL)	Elevation of Mean Sea Level	69.46	Numeric
Address	Location	Null	Alphanumeric
Date Record	Capture date	Record Date: 2021-05-20 11:11:02	Numeric
GPS Accuracy (m)	Global Positioning System (GPS) Accuracy	3.900000095	Numeric
Photo	Picture of the location	Null	Image

To clearly mark the location boundaries of patients and geographically localise them within a local government unit, the address column was further split to form an additional attribute, called the LGA. Location attributes documented as part of the datasets include Latitude, Longitude, Elevation, Date recorded, and GPS accuracy. For this publication, we were only able to provide location data for patients within the Uyo metropolis, hence, resulting in a total of 1683 MNCH records and distributed as follows: maternal=538, neonatal=720, child=425. We hope to cover other senatorial districts as soon as future funding is available.

**Figure 2.**
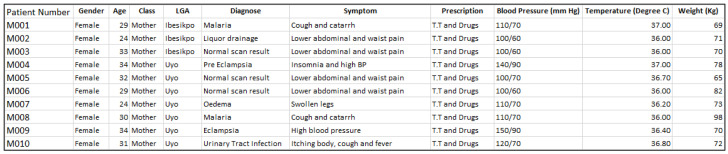
Sample maternal health dataset. LGA=local government area.

**Figure 3.**
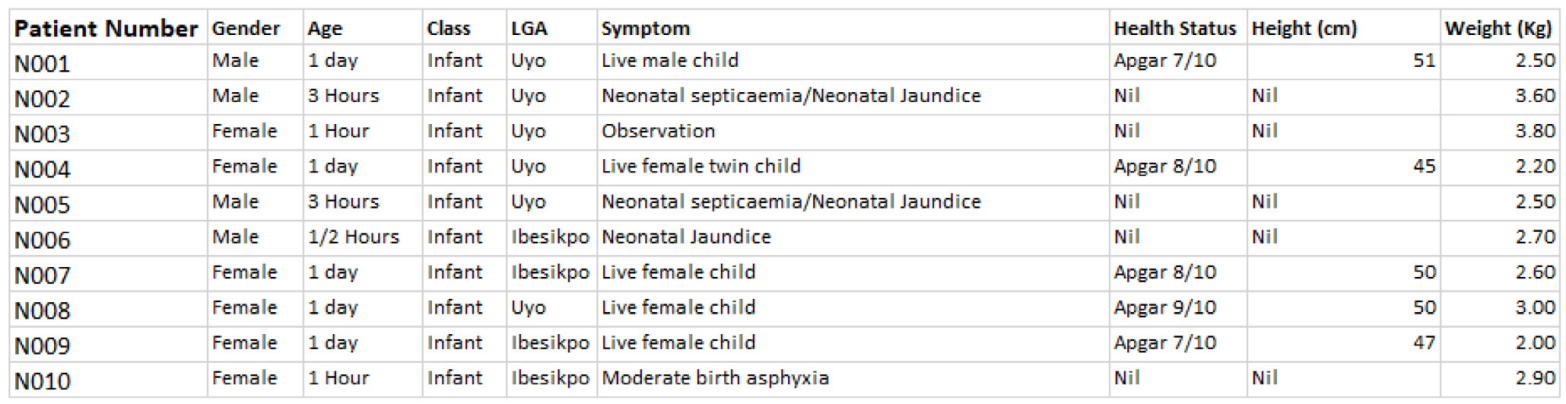
Sample neonatal health dataset. LGA=local government area.

**Figure 4.**
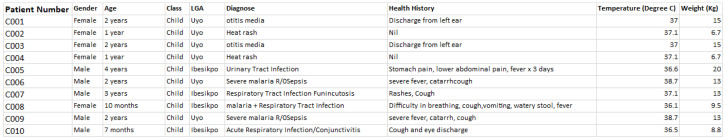
Sample child health dataset. LGA=local government area.

At the end of the data capturing exercise, the data template was converted into electronic format using Microsoft Excel, and manually merged with the geolocation records (exported CSV file) from the field (or study locations visited). The first 10 samples of the maternal, neonatal and child health datasets are given in Figure 2, Figure 3, and Figure 4, respectively. The dataset can be found as *Underlying data* ( Ekpenyong *et al.*, 2021).

## Data Availability

Open Science Framework: Maternal, Neonatal and Child Health Datasets for Spatiotemporal Data Analytics. https://doi.org/10.17605/OSF.IO/J9ZH8 ( Ekpenyong *et al.*, 2021). Data are available under the terms of the Creative Commons Attribution 4.0 International license (CC-BY 4.0). Access to restricted data (GPS data) will be made available to readers after a formal request to the corresponding author ( mosesekpenyong@uniuyo.edu.ng) and on the condition that data will be used strictly for research purposes.
