# Supplementary material for: Curating maternal, neonatal and child health (MNCH) datasets from a hospital’s catchment area in Nigeria between 2014 and 2019
Source: F1000Res. 2023 Sep 11;11:166. Originally published 2022 Feb 10. [Version 2] doi: 10.12688/f1000research.73822.2 (PMC12815018; doi:10.12688/f1000research.73822.2)
Supplement: Peer review history

# Reviewer report: Reviewer response for version 2

**DOI:** 10.5256/f1000research.155709.r433364

**Published:** 2025-12-11

**Authors:** Emmanuel Bonnet

**Affiliations:** 1 Institut de Recherche pour le Développement, CNRS Université Paris 1 Panthéon- Sorbonne, Île-de-France, France

 My assessment of this manuscript after the implementation of the previous reviewers’ recommendations, I acknowledge that substantial progress has been made between version 1 and version 2. The objectives are now clearly stated, the hypotheses have been reformulated in a more coherent and scientifically defensible manner, and the ethical safeguards regarding data anonymization and confidentiality are now described in a much more rigorous and explicit way.

 The inclusion of a location map, the enrichment of the discussion on geospatial software beyond ArcGIS alone, as well as the clarification of data availability via the OSF platform, constitute improvements that respond to the main methodological requests made by the initial reviewers.

 However, despite these advances, several important weaknesses remain unresolved and should be corrected before the manuscript can be considered fully mature. I believe that the authors, as specialists in cartography and geospatial data, could do better than providing a simple screenshot and a rough sketch for the location map. In particular, essential cartographic elements such as a scale and other mandatory components are missing.

 The most critical unresolved issue concerns GPS accuracy. Although the authors explicitly stated in their response to the first round of reviews that the numerical GPS precision range (1–9.65 metres) would be removed, this information is still present in the Methods section of version 2.

 A second major limitation concerns the spatial restriction of the dataset to the sole metropolis of Uyo. Although lack of funding is mentioned as a justification, the manuscript still does not provide a clear methodological rationale for this choice in terms of representativeness, sampling logic, or implications for the generalizability of the results.

 Although the introduction has been partially revised, it is still too long and continues to contain a substantial proportion of general statements on technology and health systems that are only indirectly related to the specific contribution of this dataset. A stronger refocusing on the originality, structure, and concrete applications of the MNCH dataset would significantly strengthen the manuscript.

 Although the temporal structure of the data is stated to be available via the OSF repository, it remains only minimally explained within the manuscript itself, even though spatio-temporal analysis is presented as one of the major intended uses of the dataset.

 In conclusion, version 2 reflects a genuine effort to take previous reviewers’ comments into account and shows notable improvements regarding the objectives, hypotheses, ethics, and data access. Nevertheless, the inconsistencies mentioned above still limit the full scientific robustness of the manuscript. A further targeted revision addressing these specific points is required to reach a level fully consistent with the standards expected for durable international indexing.

Are sufficient details of methods and materials provided to allow replication by others?

Yes

Is the rationale for creating the dataset(s) clearly described?

Yes

Are the datasets clearly presented in a useable and accessible format?

Yes

Are the protocols appropriate and is the work technically sound?

Yes

Reviewer Expertise:

Health geographer, geo-epidemiologist

I confirm that I have read this submission and believe that I have an appropriate level of expertise to confirm that it is of an acceptable scientific standard, however I have significant reservations, as outlined above.

# Reviewer report: Reviewer response for version 2

**DOI:** 10.5256/f1000research.155709.r205335

**Published:** 2023-10-16

**Authors:** Peter M. Macharia

**Affiliations:** 1 Department of Public Health, Institute of Tropical Medicine Antwerp, Antwerp, Belgium; 2 Population & Health Impact Surveillance Group, Kenya Medical Research Institute-Wellcome Trust Research Programme, Nairobi, Kenya

By a location map I meant,

1. Map out the approximate hospital catchment for which the datasets are available.
2. The map should also show other facilities in the vicinity,
3. Place the location of the map within the boundaries of the country as an inset smaller map.

Are sufficient details of methods and materials provided to allow replication by others?

Yes

Is the rationale for creating the dataset(s) clearly described?

Yes

Are the datasets clearly presented in a useable and accessible format?

Partly

Are the protocols appropriate and is the work technically sound?

Yes

Reviewer Expertise:

Geospatial health/spatial epidemiology

I confirm that I have read this submission and believe that I have an appropriate level of expertise to confirm that it is of an acceptable scientific standard.

# Reviewer report: Reviewer response for version 2

**DOI:** 10.5256/f1000research.155709.r205336

**Published:** 2023-10-16

**Authors:** Liberty Makacha

**Affiliations:** 1 School of Public Health, Imperial College London, London, England, UK; 2 Women and Children's Health, King's College London, London, England, UK; 3 Surveying and Geomatics, Midlands State University, Gweru, Midlands Province, Zimbabwe

Overall, the manuscript presents valuable data related to Maternal, Neonatal, and Child Health (MNCH) records collected from St Luke's General Hospital in Anua, Uyo, Nigeria. The dataset is aimed at facilitating research, analysis, policy formulation, and decision-making in the field of healthcare, particularly in low- and medium-income countries. The authors provide comprehensive details regarding the dataset's collection and ethical considerations. However, there are areas that still need improvement before considering it fit for indexing.

 Review with recommendations:

1. **Abstract**:
  - The abstract provides a clear and concise overview of the study, the dataset, and its purpose.
2. **Introduction**:
  - The introduction highlights the importance of MNCH data and its implications for healthcare planning effectively.
  - However, the introduction is still quite lengthy, and some parts could be condensed to maintain reader engagement.
3. **Materials and Methods**:
  - The ethical approval section is well-detailed and essential for transparency.
  - The data source and capturing procedure are clearly explained, including the steps to maintain data privacy.
  - It is unclear why the dataset collection is limited to the Uyo metropolis. The rationale for this should be clarified.
4. **Data Availability**:
  - The manuscript correctly provides information about where the dataset is available and the conditions for access.
  - The use of a Creative Commons Attribution license is appropriate for data sharing.
5. **Language and Style**:
  - The manuscript mostly follows a clear and concise writing style.
  - Some sentences are overly complex and could be simplified for better readability.
6. **Figures and Tables**:
  - Figures and tables are well-structured and contribute to understanding the text.
  - The manuscript should include more detailed explanations for certain figures, particularly Figure 1, to enhance reader comprehension.
7. **Recommendations**:
  - The introduction section needs to be streamlined, with a focus on the core objectives and research questions of the study.
  - Consider expanding the discussion on the implications and potential applications of the MNCH dataset. How can this data be used for policymaking, patient care, or research in low- and medium-income countries?
  - Clarify the reasons for focusing exclusively on the Uyo metropolis for data collection and provide a plan for potential expansion to other areas.

Overall, this manuscript presents an important dataset and research on MNCH in a low- and medium-income country context. With some revisions to improve clarity and a more concise introduction, it could be in a indexable state. Additionally, consider engaging with relevant research literature to highlight how this dataset contributes to the field.

Are sufficient details of methods and materials provided to allow replication by others?

Yes

Is the rationale for creating the dataset(s) clearly described?

No

Are the datasets clearly presented in a useable and accessible format?

Yes

Are the protocols appropriate and is the work technically sound?

Partly

Reviewer Expertise:

Geographic Information Systems, Health Geography or Spatial Epidemiology

I confirm that I have read this submission and believe that I have an appropriate level of expertise to confirm that it is of an acceptable scientific standard, however I have significant reservations, as outlined above.

# Reviewer report: Reviewer response for version 1

**DOI:** 10.5256/f1000research.77502.r173253

**Published:** 2023-05-31

**Authors:** Liberty Makacha

**Affiliations:** 1 School of Public Health, Imperial College London, London, England, UK; 2 Women and Children's Health, King's College London, London, England, UK; 3 Surveying and Geomatics, Midlands State University, Gweru, Midlands Province, Zimbabwe

The objective of this study is to create a publicly available geographically precise dataset for a data poor setting that will enable driving location sensitive decision making. The authors collected patient data and linked it to participants precise locations (home address). The article would benefit more if the authors seek grammar review from experts in the the field. Whilst the manuscript presence an important argument for data poor settings, this did not come out elaborate in the current phrasing of a bigger part of the current version of the manuscript. 

1.  ........ **Hence, knowledge of health facilities is crucial for providing informed health planning decisions.**

*The statement may need to be qualified. What knowledge of health facilities are the authors referring to here? *
2.
**.......with the urban areas holding very high concentration of patients’ population per facility.**

3.
**.....as major factors mitigating access to quality healthcare**

*I would use technical terms like patient to provider ratio to describe the same. *
4.
**mitigating factors: finance (poor living condition), increased security threat, type and nature of ailment, geography of residence, race and ethnicity, gender, age, language, and disability** . **These factors significantly determine the degree at which medical/healthcare services – including availability, timeliness, convenience, and affordability, are utilised**

*What are mitigating factors? *
5.
**Adoption of modern technology has however simplified healthcare services through the implementation of automated systems.**

*What do the authors mean by this statement? Consider rephrasing for clarity. *
6. **.....that defies real-time, patient-centred care** *. *
*Again this statement may require clarification. How does unstructured data defy patient-centred care?*
7. *.....* **and details of every parameter within the health system should be available in real-time.**

*In my opinion this statement may need to be qualified as its overly ambitious in implication for technology driven systems that aim to improve the utility of healthcare delivery systems. Most have only tended to target a subset of the core architectures that drive the same. *
8. **We demonstrate in this publication the importance of unstructured data processing to achieve semi-structured maternal, neonatal and child health (MNCH) datasets curated directly from patients’ medical file/records, to support intelligent health data mining, informed policy planning and robust decision support system design.**
*The idea behind this statement in the introduction section of the manuscript is to present the study objectives. In my opinion this objective is not elaborately presented in the current wording of the statement. Consider rephrasing. *
9.
**.......expected hypotheses on the applications of our datasets**

*What does this mean?*
10. ** MNCH datasets would encourage the development of resources for the sub-Saharan African region**
*What do the authors mean by this statement? Which resources are in discussion. Consider rephrasing. *
11.
**MNCH datasets would drive integrated GeoAI applications for robust spatiotemporal data analysis.**

*If this hypothesis is true, why has MNCH datasets failed to achieve the same all along? Consider rephrasing. *
*Do the authors still mean maternal, neonatal and child health by MNCH? If so, in my opinion I think the three hypothesis statements must be revised. MNCH datasets have ever been there both in developed and developing communities. The new argument which I thought the authors would bring is the utility of technology driven/AI powered MNCH datasets.  *
12.
**
**Data source, sample size and capturing procedure**
**

  Would the description presented here still not amount to person level data capture. SEE ---Ethical clearance was granted by the University of Uyo Health Research Ethics Committee (UNIUYO-HREC) – Ref. number: UU/CHS/IHREC/VOL.I/017 with the acceptance that the study did not require direct contact with patients.
*While the investigators would not know the the physical person behind each hospital record. It is possible that being a local community there is bound to be a level of familiarity with each other at community level. What protocols were put in place to blind the same since not individual patient consent was sought?*
13. **Furthermore, patient’s location/address was truncated by removing street number, to avoid privacy and security implication.**
*Whilst this is OK from an ethics point of view, I am of the opinion that the approach will compromise the geo-mapping utilities required by the investigators. What did the investigators do make sure that mapping was still possible using information captured in this dataset after dropping off this field. Would that amount to going back to the parent records after patient profile data capture to achieve precise location establishment at field data capture?*
14. ***Due to ethical reasons, we are only interested in the vicinity of the patient, hence the defined accuracy range.***
*1 - 9.65 metres amounts to a precise location especially in less populated communities. What is the average household to household distance in the study area? *

Are sufficient details of methods and materials provided to allow replication by others?

Yes

Is the rationale for creating the dataset(s) clearly described?

No

Are the datasets clearly presented in a useable and accessible format?

Yes

Are the protocols appropriate and is the work technically sound?

Partly

Reviewer Expertise:

Geographic Information Systems, Health Geography or Spatial Epidemiology

I confirm that I have read this submission and believe that I have an appropriate level of expertise to confirm that it is of an acceptable scientific standard, however I have significant reservations, as outlined above.

# Response

**Published:** 2023-09-05

**Authors:** Moses Ekpenyong

**Affiliations:** University of Uyo, Nigeria

Comment 1:

  ........ Hence, knowledge of health facilities is crucial for providing informed health planning decisions.

 The statement may need to be qualified. What knowledge of health facilities are the authors referring to here? 

 Response 1:

 We are referring to facilities offering MNCH services. statement is rephrased to "...Hence, knowledge of available MNCH facilities"

  Comment 2:

 .......with the urban areas holding very high concentration of patients’ population per facility.

 .....as major factors mitigating access to quality healthcare

 I would use technical terms like patient to provider ratio to describe the same.

 Response 2:

 The statement hase been rephrased to "...In urban areas, there is a notable imbalance in the patient-to-provider ratio, with a significant concentration of the patient population per healthcare facility" 

 Comment 3:

 mitigating factors: finance (poor living condition), increased security threat, type and nature of ailment, geography of residence, race and ethnicity, gender, age, language, and disability. These factors significantly determine the degree at which medical/healthcare services – including availability, timeliness, convenience, and affordability, are utilised

 What are mitigating factors? 

 Response 3:

 Mitigating factors are factors hindering access to quality healthcare. Hence the paragraph has been rephrased as:

 Oleribe et al. (2019) identified major factors hindering access to quality healthcare in sub-Saharan Africa, including financial barriers, poor governance, and limited infrastructure. In Nigeria identified hidering factors include, financial constraints due to poor living conditions, heightened security threats affecting healthcare access, the type and nature of ailments influencing healthcare choices, geographic residence impacting access, racial and ethnic disparities affecting healthcare outcomes, gender-related disparities, age-related healthcare considerations, language barriers affecting communication, and disability-related healthcare challenges. These factors significantly influence the utilization of medical and healthcare services in terms of availability, timeliness, convenience, and affordability (Babalola & Fatusi, 2009).

 Comment 4:

 Adoption of modern technology has however simplified healthcare services through the implementation of automated systems.

 What do the authors mean by this statement? Consider rephrasing for clarity. 

 Response 4:

 Statement rephrased as "The integration of modern technology into the health sector has simplified healthcare services"

 Comment 5:

 .....that defies real-time, patient-centred care. 

 Again this statement may require clarification. How does unstructured data defy patient-centred care?

 Response 5:

 The statement has been rephrased to "...defying efforts to extract meaningful insights, hinder accurate decision-making, and impede the creation of reliable healthcare solutions and strategies"

 Comment 6:

 .....and details of every parameter within the health system should be available in real-time.

 In my opinion this statement may need to be qualified as its overly ambitious in implication for technology driven systems that aim to improve the utility of healthcare delivery systems. Most have only tended to target a subset of the core architectures that drive the same. 

 Response 6:

 The sentence has been rephrased as: "To achieve this, it's essential that technology-driven systems, aiming to enhance the utility of healthcare delivery systems, prioritize the availability of real-time location-based information and comprehensive details of the healthcare system"

 Comment 7:

 We demonstrate in this publication the importance of unstructured data processing to achieve semi-structured maternal, neonatal and child health (MNCH) datasets curated directly from patients’ medical file/records, to support intelligent health data mining, informed policy planning and robust decision support system design.

 The idea behind this statement in the introduction section of the manuscript is to present the study objectives. In my opinion this objective is not elaborately presented in the current wording of the statement. Consider rephrasing. 

 Response 7:

 The statement is rephrased to distinguish the overall ojective and specific objectives:

 This publication creates a maternal, neonatal, and child health (MNCH) datasets directly sourced from patients' medical records for a data poor setting. The curated datasets are instrumental in facilitating driving location sensitive decision making, intelligent health data mining, informed policy planning, and robust decision support systems design. The specific objectives of the researh therefore include:

 To gather and compile detailed MNCH data from various sources to create a comprehensive dataset.

 To develop a standardized MNCH data and structure format.

 To convert unstructured healthcare data into a semi-structured format, making it suitable for analysis.

 To integrate location-based information into the MNCH dataset for facilitating spatiotemporal analysis and visualization.

 Comment 8:

 .......expected hypotheses on the applications of our datasets

 What does this mean?

 Response 8:

 It means the study/research hypothesis

 Comment 9:

 MNCH datasets would encourage the development of resources for the sub-Saharan African region

 What do the authors mean by this statement? Which resources are in discussion. Consider rephrasing.

 Response 9:

 Statement deleted and rephrased to address the next reviewer's comment 

 Comment 10:

 MNCH datasets would drive integrated GeoAI applications for robust spatiotemporal data analysis.

 If this hypothesis is true, why has MNCH datasets failed to achieve the same all along? Consider rephrasing. 

 Do the authors still mean maternal, neonatal and child health by MNCH? If so, in my opinion I think the three hypothesis statements must be revised. MNCH datasets have ever been there both in developed and developing communities. The new argument which I thought the authors would bring is the utility of technology driven/AI powered MNCH datasets.  

 Response 10:

 We agree with the reviewer's suggestion on change of hypothesis. The hypothesis are therefore restated as follows:

 1) Technology driven data curation practices improves MNCH outcome predictions

 2) Integration of location-based data into MNCH datasets leads to more informed policy insights and recommendations for MNCH.

 3) Geo-referenced features of MNCH datasets enables real-time demographic/spatiotemporal analysis

 Comment 11:

 Data source, sample size and capturing procedure

 Would the description presented here still not amount to person level data capture. SEE ---Ethical clearance was granted by the University of Uyo Health Research Ethics Committee (UNIUYO-HREC) – Ref. number: UU/CHS/IHREC/VOL.I/017 with the acceptance that the study did not require direct contact with patients.

 Response 11:

 The paragraphs in this section have been rephrased to clear this misconception as follows:

 The datasets used in this study originated from patients' medical records/files retrieved from St Luke's General Hospital in Anua, Uyo, Akwa Ibom State, Nigeria, which is the healthcare facility under consideration. These records cover the period from 2014 to 2019. The process of selecting properly documented files involved physically inspecting the patients' records, which were manually stored in the hospital's file cabinets or archives. To initiate this procedure, we sought informed consent through the Chief Medical Director of the hospital to obtain the necessary data. After obtaining permission, the necessary arrangements were made to commence the exercise. It's important to note that the investigators did not have direct access to the records room or the patient archives. Only files preselected by the designated officers assigned by the Chief Medical Director of the hospital were made available for the investigators' use. Before handing over these files for the capture of attributes, the assigned officers reviewed them to ensure compliance with the primary attributes of the study, as outlined in Table 1. ...

 Comment 12:

 While the investigators would not know the physical person behind each hospital record. It is possible that being a local community there is bound to be a level of familiarity with each other at community level. What protocols were put in place to blind the same since not individual patient consent was sought?

 Response 12:

 Protocols established to maintain the confidentiality and anonymity of patients' health records and to mitigate the risk of inadvertently identifying specific individuals within the local community include:

 Data De-Identification: Personal identifiers, such as names, were systematically removed or replaced with codes or pseudonyms to prevent any association between the data and specific individuals.

 Location Privacy Measures: Specific location/address details, including street numbers, were redacted or generalized to a broader geographic level to minimize the risk of identifying individual patients based on their addresses.

 Aggregated Data Analysis: Instead of analyzing individual-level data, the analysis was conducted at an aggregated or group level whenever possible. This approach ensures that results and insights do not pertain to any single person but are generalized across a broader population.

 Access Control: Access to the dataset and any related information was restricted to authorized personnel only, and strict confidentiality agreements were in place to prevent any unauthorized disclosure of patient identities.

 Ethical Review and Compliance: The study and its data-handling procedures were subject to ethical review and compliance with relevant privacy and data protection regulations and guidelines to ensure patient anonymity.

 Comment 13:

 Furthermore, patient’s location/address was truncated by removing street number, to avoid privacy and security implication.

 Whilst this is OK from an ethics point of view, I am of the opinion that the approach will compromise the geo-mapping utilities required by the investigators. What did the investigators do make sure that mapping was still possible using information captured in this dataset after dropping off this field. Would that amount to going back to the parent records after patient profile data capture to achieve precise location establishment at field data capture?

 Response 13:

 We have rephrased this in the protocol. The location (Lat and Long) records are available at OSF.

 Comment 14:

 Due to ethical reasons, we are only interested in the vicinity of the patient, hence the defined accuracy range.

 1 - 9.65 metres amounts to a precise location especially in less populated communities. What is the average household to household distance in the study area? 

 Response 14:

 Please see above. Statement deleted. Average household to household distance not established. May be useful for future works.

# Reviewer report: Reviewer response for version 1

**DOI:** 10.5256/f1000research.77502.r157440

**Published:** 2022-12-19

**Authors:** Peter M. Macharia

**Affiliations:** 1 Department of Public Health, Institute of Tropical Medicine Antwerp, Antwerp, Belgium; 2 Population & Health Impact Surveillance Group, Kenya Medical Research Institute-Wellcome Trust Research Programme, Nairobi, Kenya

Sharing such datasets is key for research. It facilitates reproducibility, generation of new research questions to improve the health of community or develop new research approaches. More important, when these datasets have linked location details, additional insights linked to space can be harnessed. I have a few thoughts which I welcome the authors to consider:

- A location map showing the approximate hospital catchment for which the datasets are available. You could also show other facilities in the vicinity, useful for context. 
- In addition to the map, provide general details/characteristics of the study area for researchers/potential users of the data but not familiar with Nigeria and the region.
- The introduction has mainly concentrated on health care access aspects of health facilities. However, not much has been said in relation to other key attributes of the data provided. That is, the data provides attributes that spans beyond a discourse of health care access only.
- ArcGIS should be given as an example of a geospatial software among others but not as the only option. Please paraphrase the sentence.
- In the abstract, you state, “The curated datasets also involved the capturing of location information from the study area, to aid spatiotemporal and informed demographic analysis”. Should it not be aid in spatial and spatio-temporal analysis?
- As these datasets were refer to between 2014 and 2019, and one envisioned application will be spatio-temporal analysis, a date attribute should be availed on the main database. You can truncate it monthly or quarterly in each year to align with confidentiality guidelines.
- I wonder whether the tittle could be edited to Maternal, neonatal and child health (MNCH) datasets from a hospital’s catchment area in Nigeria between 2014 and 2019?

Are sufficient details of methods and materials provided to allow replication by others?

Yes

Is the rationale for creating the dataset(s) clearly described?

Yes

Are the datasets clearly presented in a useable and accessible format?

Partly

Are the protocols appropriate and is the work technically sound?

Yes

Reviewer Expertise:

Geospatial health/spatial epidemiology

I confirm that I have read this submission and believe that I have an appropriate level of expertise to confirm that it is of an acceptable scientific standard, however I have significant reservations, as outlined above.

# Response

**Published:** 2023-09-05

**Authors:** Moses Ekpenyong

**Affiliations:** University of Uyo, Nigeria

Comment 1:

 Sharing such datasets is key for research. It facilitates reproducibility, generation of new research questions to improve the health of community or develop new research approaches. More important, when these datasets have linked location details, additional insights linked to space can be harnessed. I have a few thoughts which I welcome the authors to consider:

 A location map showing the approximate hospital catchment for which the datasets are available. You could also show other facilities in the vicinity, useful for context. 

 Response 1:

 Reviewer's comment is addressed and captured as Fig. 1.

 Comment 2:

 In addition to the map, provide general details/characteristics of the study area for researchers/potential users of the data but not familiar with Nigeria and the region.

 Response 2:

 Reviewer's comment addressed. general details/characteristics of the study area provided as follows:

 St Luke’s General Hospital, Anua is one of the foremost Missionary Hospitals in the South-South and South-East Nigeria. The hospital is located along Nwaniba Road in Uyo Metropolis of Akwa Ibom State, Nigeria, West Africa. A location map showing the approximate hospital catchment for which the datasets are available is presented in Fig. 1.

 Comment 3:

 The introduction has mainly concentrated on health care access aspects of health facilities. However, not much has been said in relation to other key attributes of the data provided. That is, the data provides attributes that spans beyond a discourse of health care access only.

 Response 3:

 Other key attributes of the data as provided in Tables 1 and 2 and are well documented and explained in the OSF, as prescribed by the F1000Research.

 Comment 4:

 ArcGIS should be given as an example of a geospatial software among others but not as the only option. Please paraphrase the sentence.

 Response 4:

 Sentence paraphrased and enriched as follows:

 Geospatial software tools for managing and visualizing MNCH datasets are discussed below:

 ArcGIS: Developed by Esri (Environmental Systems Research Institute) is a comprehensive and widely used GIS software suite, designed for managing, analyzing, visualizing, and sharing geospatial data and information.

 QGIS (Quantum GIS): An open-source and user-friendly GIS software that provides powerful mapping and data analysis capabilities.

 GRASS GIS (Geographic Resources Analysis Support System): An open-source GIS software that focuses on geospatial data management, analysis, and visualization.

 R with Spatial Packages: The R programming language, coupled with specialized spatial packages like "sp," "sf," and "raster," can be used for geospatial data analysis and visualization.

 GeoDa: A free software program designed for exploratory spatial data analysis, spatial statistics, and spatial econometrics.

 SAGA GIS: An open-source geographic information system with a wide range of geospatial analysis and modeling tools.

 Mapbox: A cloud-based platform for designing and publishing custom maps, providing tools for geospatial data visualization and analysis.

 OpenStreetMap (OSM) Tools: Tools and APIs for working with OpenStreetMap data, which can be useful for incorporating community-contributed geographic data.

 Google Earth Engine: A cloud-based platform for analyzing and visualizing Earth observation data, which can be valuable for spatiotemporal analysis.

 The choice of geospatial software however depends on factors such as, data requirements, project goals, budget, and end-users’ familiarity. Each of these software options offers unique features and capabilities for working with geospatial data.

 Comment 5:

 In the abstract, you state, “The curated datasets also involved the capturing of location information from the study area, to aid spatiotemporal and informed demographic analysis. Should it not be aid in spatial and spatio-temporal analysis?

 Response 5:

 Yes, statement rephrased to '...facilitating spatiotemporal analysis'

 Comment 6:

 As these datasets were refer to between 2014 and 2019, and one envisioned application will be spatio-temporal analysis, a date attribute should be availed on the main database. You can truncate it monthly or quarterly in each year to align with confidentiality guidelines.

 Response 6:

 The date attribute is revealed in the OSF platform. 

 Comment 7:

 I wonder whether the tittle could be edited to Maternal, neonatal and child health (MNCH) datasets from a hospital’s catchment area in Nigeria between 2014 and 2019?

 Response 7:

 We agree with reviewer's comment. Appears very informative. Hence, we have modified the title to: "Curating Maternal, neonatal and child health (MNCH) datasets from a hospital’s catchment area in Nigeria between 2014 and 2019".
